# Cytomegalovirus infection in a splenectomized with *β*‐thalassemia major: immunocompetent or immunosuppressed?

**DOI:** 10.1002/ccr3.1001

**Published:** 2017-05-16

**Authors:** George D. Liatsos, Maria Pirounaki, Angelina Lazareva, Georgia Kikezou, Spyridon P. Dourakis

**Affiliations:** ^1^ ^nd^2^nd^ Academic Department of Internal MedicineMedical SchoolHippokratio General HospitalNational and Kapodistrian Athens UniversityAthensGreece

**Keywords:** Cytomegalovirus, immunosuppression, pneumonia, splenectomy, *β*‐thalassemia

## Abstract

We should possibly revise our knowledge about risk assessment of splenectomized individuals with *β*‐thalassemia major. Besides their known risk of certain bacterial infection, they might be also in a risk of life‐threatening primary cytomegalovirus (CMV) infection and end‐, multi‐organ disease, in the context of their immunosuppression status. Prompt and appropriate treatment initiation can be life saving.

A 40‐year‐old Caucasian splenectomized female with *β*‐thalassemia major regularly transfused with packed and leukocyte‐depleted red blood cells (RBC), presented acutely ill with fever, dry cough, and mild shortness of breath. Auscultation revealed bilateral diffuse fine crackles. Routine laboratory findings are shown on Table 1; thorax CT revealed bilateral interstitial lung infiltrates and small nodules marked toward the lower lobes, with a few ground‐glass areas and bilateral pulmonary effusions. She was initially treated as a possible community‐acquired pneumonia (Levofloxacin, Piperacillin‐Tazobactame, and Oseltamivir), and she required supplemental oxygen therapy. Extended work‐up was conducted in order to define the causative agent (Table 1). During hospitalization patient remained febrile with radiographic progression. On day 7, laboratory reported a positive rt‐PCR for cytomegalovirus (CMV) from a blood specimen obtained on day 2. Due to reported vision abnormalities a fundoscopy revealed retinitis of her left eye, and she was subjected to bronchoscopy; BAL examination was unremarkable for other respiratory pathogens except for positive rt‐PCR for CMV. In addition, serum re‐evaluation revealed a tenfold increase in IgG and a threefold increase in IgM titers.

**Table 1:** Patient's laboratory test results during hospitalization and follow‐up

Blood test	Admission	Day 7	Day 14	Day 16 Discharge	Two months later
WBC count/*μ*L	12,340	19,570	13,620	10,910	8670
Neutro %	55	33	36	41	51
Lympho %	24	50	53	51	46
Monocytes %	6	4	5	4	2
Atypical forms %	12	10	3	1	0
Hct %	36	33.5	28.2	35.5	34.3
Hb g/dL	11.9	10.7	10.2	11.3	11.1
PLT count/*μ*L	422,000	465,000	539,000	410,000	395,000
Urea mg/dL	61	51	75	81	52
Creatinine mg/dL	1.5	1.5	1.4	1.5	1.2
LDH IU/L	423	492	474	367	255
AST IU/L	150	76	70	59	32
ALT IU/L	163	88	39	34	22
*γ*‐GT IU/L	504	460	345	387	108
ALP IU/L	311	370	332	335	165
TBIL mg/dL	1	0.8	1	0.9	0.8
pH	7.30	7.37	7.41	7.37	n.a.
pO_2_ mm/Hg	49	56	69	105	n.a.
pCO_2_ mm/Hg	33	32	38	32	n.a.
HCO_3_^‐^ mmol/L	17.5	20.1	24.5	20.2	n.a.
Anti‐CMV IgG^a^ IU/mL	13.3 gray zone	123 pos.	144 pos.	n.a.	n.a.
Anti‐CMV IgM^a^ AU/mL	14.8 neg.	46.4 pos.	52.3 pos.	n.a.	n.a.
Anti‐HIV _1,2_	Negative	–	–	–	–
HBsAg anti‐HCV	Negative Negative	–	–	–	–
VCA EBV‐G	240 pos.	–	–	–	–
VCA EBV‐M	<10 neg.	–	–	–	–
HSV 1,2 G	17.9 pos.	–	–	–	–
HSV 1,2 M	<0.5 neg.	–	–	–	–
Toxo‐G2 ‐M	<3.0 neg	–	–	–	–
VZV IgG	2301 pos.	–	–	–	–
VZV IgM	<0.10 neg.	–	–	–	–
Rest of laboratory evaluation for other pathogens^b^	Negative	–	–	–	–
Blood rt‐PCR for CMV detection	1.2 × 10^4^ copies/mL	–	–	Negative	–
BAL rt‐PCR for CMV detection	–	Positive	–	–	–
Samples from previous transfusions^c^ (rt‐PCR for CMV detection)	–	–	Four blood units all negative		
Flow cytometric analysis of blood T‐lymphocytes^d^	–	–	CD_4_^+^ T‐lymphocytes 700/*μ*L	–	–
BAL analysis cells (<13 × 10^4^/mL)	–	34.75 × 10^4^	–	–	–
BAL smear (%) (normal range)	–	Macrophages 44% (>84)	–	–	–
BAL smear (%) (normal range)	–	Lymphocytes 55% (10–15)	–	–	–
BAL lymphocytes cytometry^e^	–	–	–	–	–
B‐cell (CD20) % (normal range)	–	4 (0–12)	–	–	
T‐cell (CD3) % (normal range)	–	92 (63–88)	–	–	–
T‐help (CD4) % (normal range)	–	32 (36–70)	–	–	–
T‐suppr (CD8)% (normal range)	–	60 (20–40)	–	–	–
Thelp/Tsuppr ratio (normal range)	–	0.53 (0.9–2.5)	–	–	–
NK cells (CD57) % (normal range)	–	4 (2–14)	–	–	–

a CMV antibodies determined one week and ten months before admission were negative (titers<5.0).

b Blood, urine cultures all negative; antibody detection against *Brucella*,* Leptospira*,* Coxiella burnetti*,* Rickettsia conorii*,* Legionella pneumophila*,* Mycoplasma pneumoniae*,* Chlamydia pneumoniae,* and *psittaci* were all negative.

c Stored blood samples obtained from the transfusion units of patient's last two transfusion courses covering a time interval of almost 40 days before disease onset. Thus, the acquisition of the virus was community or sexual transmission.

d CD_3_^+^ 52.4% (60–85), T‐cytotoxic (CD_3_^+^ CD_8_^+^) 39.2% (9–39), T‐helper (CD_3_^+^, CD_4_^+^) 13.2% (29–55), ratio (CD_4_/CD_8_) 0.34 (1–3.4), absolute lymphocyte count 5308/*μ*L; these results are in agreement with observed transient T‐cell abnormality during CMV infection in splenectomized cases.

e Findings absolutely consistent with T‐cell abnormality observed in patient's blood.

Considering all together, medical history, clinical, and laboratory findings, the diagnosis of life‐threatening primary CMV infection with multi‐organ involvement (lungs, retina, and liver) was established. Ganciclovir 137 mg *iv* was introduced (Induction dose: 2.5 mg/kg of body weight qd, as adjusted to her renal failure GFR 25–49 mL/min), and was administered for a week followed by valganciclovir 450 mg *per os* (Maintenance dose: 450 mg qd as adjusted to her renal failure GFR 40–59 mL/min) for three more weeks. Fever resolved within 3 days from ganciclovir initiation, whereas pulmonary function and fundoscopic findings gradually improved by the end of the ganciclovir treatment. Close follow‐up for the next 4 months was uneventful for relapse.

Several authors described cases of severe primary CMV infections in immunocompetent adults comprising pneumonia, hepatitis, retinitis, encephalitis, myocarditis, orchitis, pancreatitis, and multi‐organ involvement. Mortality in case series is reported high, especially in those with non‐CNS disease, and/or multi‐organ involvement 1, as in our case. The spleen provides first‐line filtration of, and the defense against the virus 2. The susceptibility to CMV is considered to be due to impaired cell‐mediated immunity, and reduction in levels of IgM 3. The splenomegaly detected in patients with CMV infection along with reported cases of spontaneous splenic rupture with the identification of CMV inclusions in it 2, suggests that the spleen is a replication site of and, hence, organ of early immunity against CMV infection. In addition, primary CMV infection elicits an early strong IgM response 4. Particularly, human blood IgM memory B cells are circulating splenic marginal zone B cells, and splenectomized people have undetectable IgM memory B cells. The inadequate IgM production results in prolonged CMV illness and elicits overwhelming IgG and cytotoxic T‐cell responses, also measured in our case (in both blood and BAL), and are evidenced by marked large granular lymphocyte proliferation and TCR*γ* gene rearrangements 4. The adequate function and quality of cytotoxic T‐cells seems to be responsible for the ultimate control in CMV circulation 4.

Red blood cells transfusions in *β*‐thalassemia major on the other hand lead to iron overload which is considered responsible for several immunological abnormalities including defective phagocytic and chemotaxis activity of macrophages and neutrophils, alteration in T and B lymphocytes subsets and function, as well as alteration in cytokines responses 5. The serum levels of TGF‐*β* and IL‐23 are significantly increased in patients with *β*‐thalassemia major, suggesting an inflammatory status associated with the suppression of T‐cell immune response, while at the same time they show a stimulated phenotype 5. Furthermore, transfusion‐induced upregulated expression of inhibitory receptor NKG2A on circulating NK cells in those patients has a close association with the decreased cytotoxicity of peripheral NK cells 6. Of notice, our patient suffered secondary hemochromatosis (serrum ferittin levels 14,599ng/mL on admission, and high ferrum deposition on liver MRI) due to her poor compliance to chelation treatment.

The diagnosis of acute CMV infection in our case was based on serology, which established seroconversion. The relative slow and mild IgM productions, compared to IgG, are in accordance with reports of other splenectomized cases, that IgM response is impaired 4, whereas IgG response is augmented and antedated. Flow cytometric analysis of the blood and BAL revealed relative high T‐cytotoxic, low T‐helper, and low CD4/CD8 ratio (Table 1), in agreement with the observation of transient T‐cell abnormality occurring during CMV infection in other splenectomized cases.

Despite the absence of guidelines, several authors suggest that the treatment of severe primary CMV infection in immunocompetent cases may be of benefit or even life saving, mostly in those with pneumonia or multi‐organ involvement 1, 2 as in our case. When CMV diagnosis was established, ganciclovir was introduced, and patient improved by the end of its course.

We present the first reported case of severe, primary multi‐organ CMV infection in a splenectomized patient with *β*‐thalassemia major. The issue of considering patients with that medical history among the traditional immunosuppressed groups in danger for severe CMV infection requires further intention. Therefore, we could possibly revise our knowledge about risk assessment of splenectomized individuals with *β*‐thalassemia. Besides their serious risk of certain bacterial infection, they might be also in a risk of life‐threatening primary CMV infection.

## Authorship

GDL: consultant internist, responsible for the hospitalization and outpatient follow‐up of the case, and also had the major role in writing the manuscript. MP: consultant in infectious diseases, responsible for the hospitalization and outpatient follow‐up of the case. AL: resident doctor, responsible for patient's hospitalization. GK: resident doctor, responsible for patient's hospitalization. SD: Professor of Internal Medicine and Hepatology, Director of the 2^nd^ Academic Department of Internal Medicine; had the whole supervision of the case. All authors had access to the data and a role in writing the manuscript.

## Conflict of Interest

No one of the authors of the present article have any conflicts of interest to declare.

## References

[1] Eddleston, M. , S. Peacock , M. Juniper , and D. A. Warrel . 1997 Severe cytomegalovirus infection in immunocompetent patients. Clin. Infect. Dis. 24:52–56. 8994755 10.1093/clinids/24.1.52

[2] Alliot, C. , C. Beets , and M. Besson . 2001 Spontaneous splenic rupture associated with CMV infection: report of a case and review. Scand. J. Infect. Dis. 33:875–877. 11760178 10.1080/00365540110027114

[3] Baumgartner, J. D. , M. P. Glauser , A. L. Burg‐black , R. D. Black , N. Pyndiah , and R. Chiolero . 1982 Severe cytomegalovirus infection in multiply transfused splenectomized trauma patients. Lancet 2:63–66. 6123807 10.1016/s0140-6736(82)91688-9

[4] Han, X. Y. , P. Lin , H. M. Amin , and A. Ferrajoli . 2005 Postsplenectomy cytomegalovirus mononucleosis. Marked lymphocytosis, TCR*γ*gene rearrangements, and impaired IgM response. Am. J. Clin. Pathol. 123:612–617. 15743745 10.1309/HLBB-K8V0-A6T8-BYV8

[5] Balouchi, S. , M. Gharagozloo , N. Esmaeil , M. Mirmoghtadaei , and B. Moayedi . 2014 Serum levels of TGF‐*β*, IL‐10, IL‐17, and IL‐23 cytokines in *β*‐thalassemia major patients: the impact of silymarin therapy. Immunopharmacol. Immunotoxicol. 36:271–274. 24945737 10.3109/08923973.2014.926916

[6] Zou, Y. , Z. X. Song , Y. Lu , X. L. Liang , Q. Yuan , S. H. Liao , et al. 2016 Up‐regulation of NKG2A inhibitory receptor on circulating NK cells contributes to transfusion‐induced immunodepression in patients with *β*‐thalassemia major. J. Huazhong Univ. Sci. Technol. Med. Sci. 36:509–513. 27465324 10.1007/s11596-016-1616-5

